# Electrochemical Wiring of a Metal Nanofilament to Form a Molecular Junction

**DOI:** 10.1021/acs.jpclett.6c00582

**Published:** 2026-05-25

**Authors:** Sekito Nishimuro, Tohru Tsuruoka, Tatsuhiko Ohto, Koya Akashi, Tomoaki Nishino, Kazuya Terabe, Satoshi Kaneko

**Affiliations:** † Department of Materials Science and Engineering, School of Materials and Chemical Technology, 13290 Institute of Science Tokyo, 2-12-1, Ookayama, Meguro-ku, Tokyo 152-8550, Japan; ‡ Research Center for Materials Nanoarchitectonics (MANA), National Institute for Materials Science (NIMS), 1-1 Namiki, Tsukuba, Ibaraki 305-0044, Japan; § Graduate School of Engineering, 12965 Nagoya University, Furo-cho, Chikusa-ku, Nagoya 464-8603, Aichi, Japan; ∥ Department of Chemistry, School of Science, 13290 Institute of Science Tokyo, 2-12-1, Ookayama, Meguro-ku, Tokyo 152-8550, Japan

## Abstract

Leveraging the functionalities of molecules in electronic devices seems promising for the development of next-generation technologies. However, wiring an electrode to a molecular layer is challenging. Here, we introduce a method for connecting a microscale metal electrode to a molecular layer using an atomic switch that controls the formation and rupture of a metal nanofilament using a bias voltage. Specifically, a 1,4-benzenedithiol (BDT) molecular layer is embedded within an atomic switch. A bias sweep applied to the Ag top electrode induces a transition between a high-conductive state (2 m*G*_0_, where *G*_0_ = 2*e* ^2^/*h*) and a low-conductive state (35 μ*G*_0_), with both states exhibiting nonvolatile operation. Current–voltage analysis and density functional theory simulation reveal that a Ag/BDT/Ag junction with approximately 20 molecules forms in the high-conductive state. Thus, our method is effective for the wiring of a microscale electrode to a nanoscale molecular layer.

## Graphical abstract

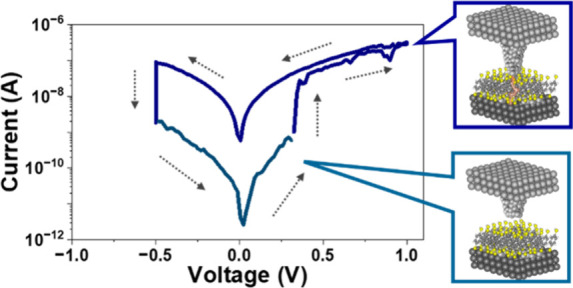

Organic molecule-based devices are promising contributors to energy efficiency toward a sustainable society.^1–3^ The versatility of various functionalities of organic molecules is leveraged in light-emitting diodes,^4,5^ photovoltaics,^6,7^ and flexible electronics.^8,9^ Nanoscale fabrication of organic devices offers numerous advantages, including high-density accumulation, multifunctionality, and portability.^10–12^ Recent research demonstrates the electron transport of single molecules, unveiling distinctive properties such as rectification^13^ and switching.^14^ Advanced wiring and integration techniques must be developed to harness these functionalities. An atomic switch, which controls the redox reaction and diffusion of a metal ion in a solid electrolyte,^15^ is a promising solution for wiring and integration, as it can facilitate the formation of nanoscale filaments using an applied bias voltage. Moreover, considering the potential use of atomic switches in memristors, field-programmable gate arrays, and neuromorphic network computing, component integration is expected to yield multifunctional devices with low energy consumption.^16–18^ A novel method for forming acetylene molecular junctions was recently reported.^19^ Recent findings reveal a connection between an acetylene molecule trapped in a Ta_2_O_5_ thin film and a Ag filament.^19^ The next step is the development of devices that incorporate molecular layers, which will promote the operation reliability and ease of handling. However, the wiring of a nanofilament to an embedded molecular layer through an atomic switch has yet to be demonstrated.

This study demonstrates nanoscale wiring to a self-assembled monolayer (SAM), based on atomic switch operation. We fabricated atomic switches embedded with 1,4-benzenedithiol (BDT), which is the model system of the molecular junction.^20–23^ The current–voltage (*I*–*V*) characteristics of the atomic switch with the BDT layer exhibited nonvolatile behavior when switching between a low-conductive (L) state and a high-conductive (H) state. Calculations based on density functional theory (DFT) with metal/BDT/metal structures suggested that BDT made homogeneous contact with Ag (Ag/BDT/Ag) rather than inhomogeneous contact with Pt (Pt/BDT/Ag). Furthermore, we analyzed the *I*–*V* curves using a tunneling model to characterize the junction structure and electronic properties in the H state. The analysis yielded the filament cross-section, tunnel barrier height, and tunneling distance, supporting electron transport via the BDT monolayer.

The atomic switch with the BDT layer was fabricated following the fabrication process of atomic switches.^19,24,25^ First, the bottom electrode was deposited via the electron-beam deposition of 5 nm-thick Ti and 35 nm-thick Pt on a Si substrate covered with 200 nm-thick SiO_2_ (Figure 1(a)). Then, the substrate was immersed in a 100 μM ethanol solution of BDT for 16 h and then rinsed using ethanol to form BDT SAMs on the bottom Pt electrode (Figure 1(b)). Next, an 8 nm-thick Ta_2_O_5_ layer, serving as a metal ion-conducting electrolyte, was deposited via radio frequency sputtering from polycrystalline targets using a gas mixture of 77% Ar and 23% O_2_ (Figure 1(c)). Finally, 20 nm-thick Ag and 30 nm-thick Pt were electron-beam deposited as the top electrode and coating layer, respectively (Figure 1(d)). The coating layer was intended to protect the Ag electrodes from oxidation.^25,26^ Each atomic switch on the substrate had a cross-point structure with a junction area of 5 × 5 μm^2^ (Figure 1).

**Figure 1:**
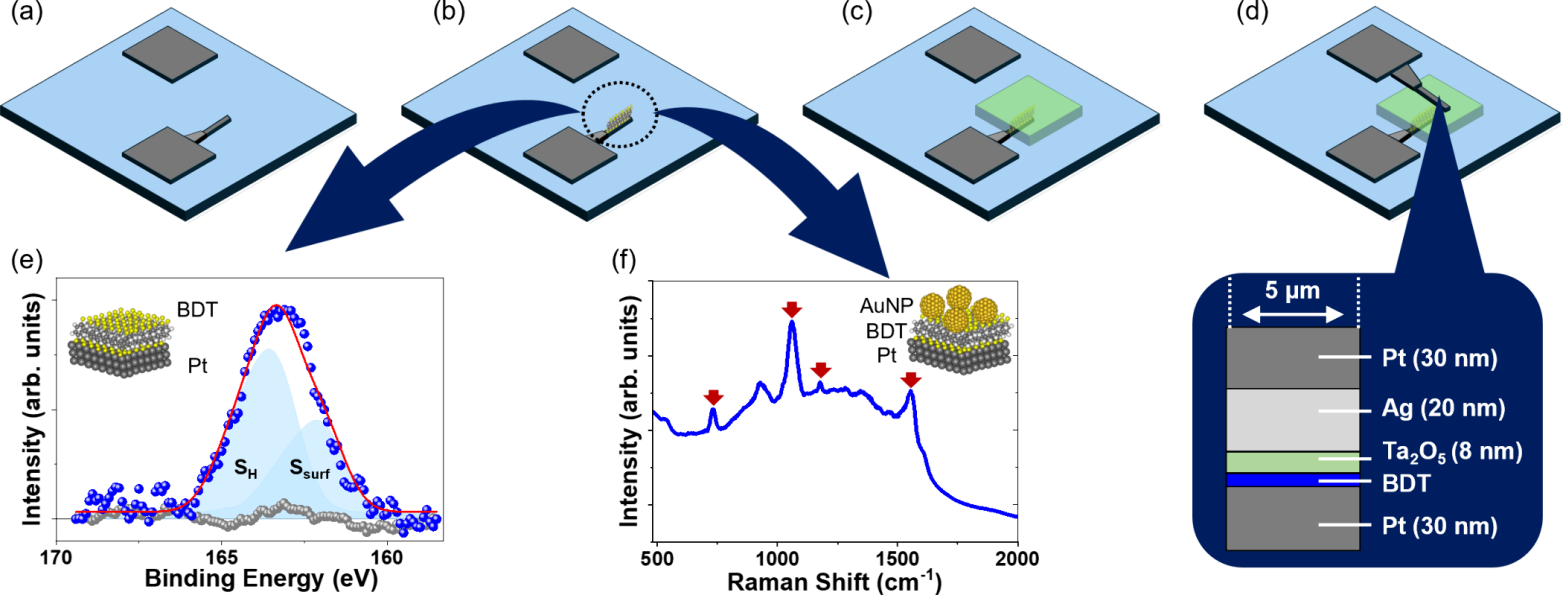
Fabrication of atomic switch incorporating BDT monolayer. (a) Fabrication of bottom electrode. (b) Fabrication of BDT SAMs. (c) Deposition of solid electrolyte layer. (d) Fabrication of top electrode. Inset: cross-section of fabricated atomic switch. (e) XPS spectra of Pt-covered Si substrates with (blue dots) and without (gray dots) BDT layer. (f) SERS spectrum of Au nanoparticle (AuNP)/BDT on Pt/Si substrate.

The presence of BDT on the bottom electrode was examined through X-ray photoelectron spectroscopy (XPS) and surface-enhanced Raman scattering (SERS). Figure 1(e) shows the S 2p XPS spectra obtained from the Pt-covered Si substrates with BDT (blue dots) and without BDT (gray dots). A broad peak was observed at approximately 163.3 eV for the Pt electrode with BDT. This peak could be decomposed into two components using two Gaussian functions, embedding the peak splitting caused by spin–orbit interactions.^27,28^ The above spectral deconvolution also showed two peaks at 162.0 ± 0.4 and 163.4 ± 0.2 eV, originating from the sulfur directly connected to the Pt surface (S_surf_) and thiol (S_H_) (details are provided in Supporting Information S1).^27–32^ In the SERS spectrum, characteristic vibrational modes were observed at 731, 1061, 1178, and 1557 cm^–1^ (red arrows in Figure 1(f)). These peaks were assigned to the vibrational modes of BDT: ν_7a_ (C–S stretching mode), ν_1_ (ring breathing mode), ν_9a_ (C–H bending mode), and ν_8a_ (C=C stretching vibrational mode), respectively (detailed information about the experimental conditions and vibrational assignment are provided in Supporting Information S2).^33,34^ The XPS and SERS spectra verify the formation of the BDT layer on the Pt electrodes.

*I*–*V* measurements were performed at room temperature under ambient conditions. The bias voltage was swept to the Ag top electrode while the Pt bottom electrode was grounded. The typical *I*–*V* curves of the atomic switches with and without BDT are shown in Figure 2(a). Without the BDT layer (gray curve), the current abruptly increased to the compliance value (100 μA) at 0.5 V in the positive bias sweep, which was set by the utilized source measure unit. This corresponded to the SET process to the ON state and was attributed to the formation of a metal filament between the Ag and Pt electrodes. For the negative bias sweep, the atomic switch exhibited the RESET process to the OFF state at −0.2 V, which corresponded to the rupture of the generated metal filament. This is the typical behavior of gapless atomic switches having thin oxide films as the solid electrolyte (Supporting Information S3).^25,35^ During the SET process, Ag atoms from the Ag electrode oxidized to the positive ion form (Ag^+^) and migrated toward the Pt electrode under the voltage bias. When the Ag ion concentration exceeded the threshold, a Ag filament formed between the Ag and Pt electrodes in the Ta_2_O_5_ layer. In the subsequent RESET process, the Ag filament was ruptured by thermochemical reactions, such as the oxidation of Ag atoms on the filament and Joule heating.^36^

**Figure 2:**
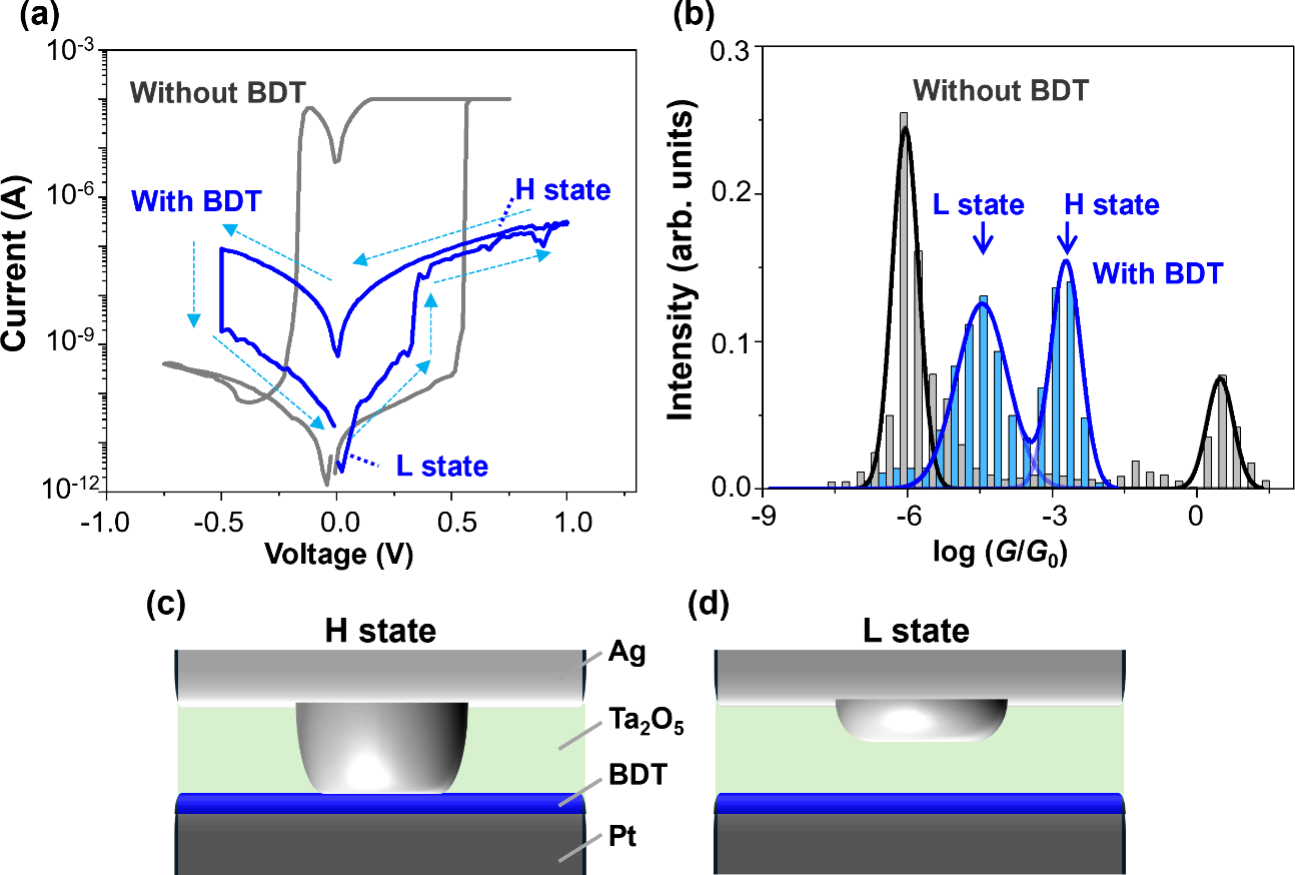
(a) *I*–*V* curves of atomic switches with (blue curve) and without (gray curve) BDT layer. The dashed arrows represent the direction of the current trajectory. (b) Histograms of conductance during operation of atomic switches with (blue bars) and without (gray bars) BDT layer, constructed from 263 and 44 *I*–*V* curves for the atomic switches with and without the BDT layer, respectively. The counts were normalized by the total number of data points. Schematic images of (c) H state and (d) L state of the atomic switch with the BDT layer.

Although the atomic switch with the BDT layer exhibited similar switching behavior, its ON and OFF states differed from those of the atomic switch without the BDT layer. Under the positive bias sweep, the current abruptly increased at 0.3 V but did not reach the compliance value, remaining at tens of nanoamperes (blue curve). In the negative bias sweep, this current level was maintained, showing nonvolatile behavior, and then dropped at −0.5 V. The resistance of the ON (OFF) state of the atomic switch with the BDT layer was higher (lower) than that of the atomic switch without the BDT layer. To distinguish between the ON and OFF states of atomic switches, those of the atomic switch with BDT are hereafter referred to as the high-conductive (H) state and low-conductive (L) state, respectively.

The conductance histograms in Figure 2(b) are in the unit of *G*_0_, which corresponds to the conductance of a single atomic point contact, given by 2*e* ^2^/*h*, where *e* is the elementary charge and *h* is the Planck constant. These histograms were constructed from the measured *I*–*V* curves of the atomic switches with (blue bars) and without (gray bars) the BDT layer. The ON and OFF states of the atomic switch without the BDT layer had conductance values of 3 *G*_0_ and 1 μ*G*_0_, respectively. ON states exceeding 1 *G*_0_ suggested the formation of a metal filament between the Ag and Pt electrodes. The conductance values of the H and L states of the atomic switch with the BDT layer were 2 m*G*_0_ and 35 μ*G*_0_, respectively. The smaller conductance of the H state suggested that the atomic switch with the BDT layer had a metastable state, unlike in the case of the ON state of the atomic switch without the BDT layer. The conductance of the H state (2 m*G*_0_) was in the regime of molecular conductance.^37–42^ The obtained conductance value was approximately one-tenth that of a Pt/BDT/Pt single-molecule junction.^41^ In the analogy of the nonvolatile behavior of the atomic switch without the BDT layer and considering the conductance of organic monolayers, the H state of 2 m*G*_0_ was attributed to the state in which a Ag filament connected to the BDT layer (Figure 2(c)). The L state corresponded to the state where the metal filament ruptured, forming a gap between it and the BDT layer (Figure 2(d)).

To understand the microscopic structure of the metal filament connected to the BDT layer, we combined the nonequilibrium Green’s function method with DFT calculations on the molecular junction with three configurations (Figure 3(a)). The junction consisted of a single BDT molecule connected to electrode 1 (El 1) and electrode 2 (El 2) on opposite sides, with different material combinations: Ag/Ag, Pt/Pt, and Pt/Ag (Supporting Information S5). Given the swept bias region for the *I*–*V* curves, the Ta_2_O_5_ bandgap was large enough to have a negligible contribution to transport.^43^ Figure 3(b) plots the simulated transmission curves for each electrode as a function of its energy relative to the Fermi energy. In discussing the contribution of the metal species to the electron transport, we focus on the states at the Fermi energy. Large transmission probabilities were obtained for the Pt/BDT/Pt and Pt/BDT/Ag junctions. Such large probabilities were attributed to the large local density of states at the Fermi level for the Pt surface.^37–42,44,45^ A summation of the transmission probability of each channel at the Fermi energy provided conductance values of 20 m*G*_0_, 0.3 *G*_0_, and 0.19 *G*_0_ for the Ag/BDT/Ag, Pt/BDT/Pt, and Pt/BDT/Ag electrodes, respectively (Table 1). The large conductance in the BDT layer connected to the Pt electrode also arose from the large local density of states near the Fermi level for the Pt surface.^37–42^

**Figure 3:**
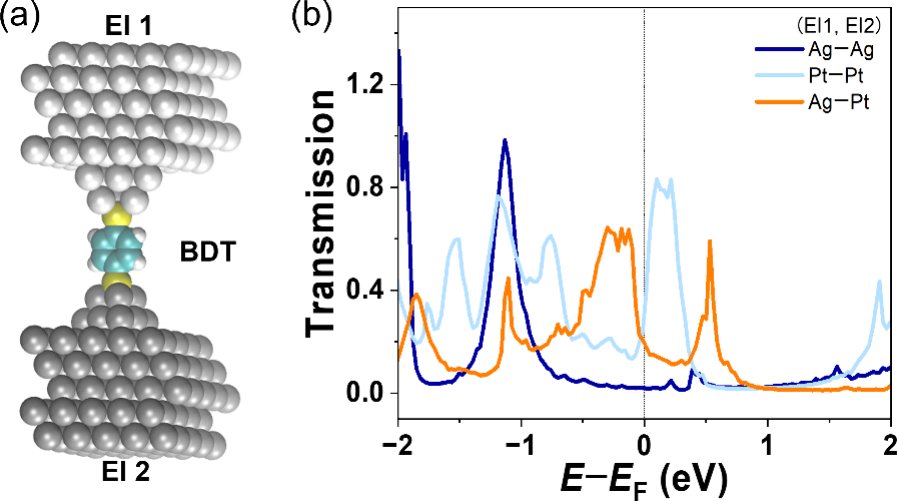
(a) Structural model for transmission calculation. (b) Transmission curves for BDT single-molecule junctions: Ag/BDT/Ag junction (dark blue), Pt/BDT/Pt junction (light blue), and Ag/BDT/Pt junction (orange).

**Table 1:** Conductance at the Fermi Energy

El 1	Ag	Pt	Ag
El 2	Ag	Pt	Pt
Conductance (*G*_0_)	2 × 10^–2^	0.3	0.19

Therefore, the conductance of the Ag/BDT/Pt heterojunction significantly exceeded that of the Ag/BDT/Ag homojunction. However, the conductance of the H state in the atomic switch with the BDT layer (2 m*G*_0_) was much smaller than that of the Pt/BDT/Pt and Ag/BDT/Pt junctions and was closer to that of the Ag/BDT/Ag junction. Furthermore, given that the experimentally measured conductance of the Pt/BDT/Pt junction (0.03 *G*_0_) was smaller than the theoretically calculated value, the H state is considered to originate from the Ag/BDT/Ag junction. Considering the mechanism of filament formation and rupture, the formation of the Ag/BDT/Ag junction may be reasonable. According to the electrochemical nucleation theory,^25,36,46^ nucleation occurs on a Pt electrode when the Ag concentration exceeds a threshold. In previous studies, energy-dispersive X-ray spectroscopy revealed the presence of metal originating from the top electrode within the bottom electrode of an atomic switch.^47,48^ Even for an SAM-covered Pt electrode, Ag is expected to deposit onto the Pt electrode during the electrochemical process, through either the percolation of Ag ions through the SAM^49–52^ or electrochemical precipitation at the SAM defects.^53–55^ Ag clusters persist on the Pt surface after a series of atomic switch operations. These abundant Ag clusters on the Pt surface allow BDT to migrate to the Ag surfaces,^56–58^ thereby connecting the Ag clusters with BDT. The presence of Ag clusters on the Pt electrode was further supported by conductance evolution (Supporting Information S4): the conductance of the L state increased relative to the initial condition, suggesting the presence of residual Ag clusters.^24,36^ Consequently, the Ag/BDT/Ag junction may have formed and stabilized during switching cycles.

Here, we first examine the overall shape of the *I*–*V* response to assess the contact geometry of the junction in the H state (Figure 4(b)). Because the interfacial geometry strongly affects electron transport, analysis of *I*–*V* characteristics can provide nondestructive in situ insights into the interfacial structure. This approach, albeit indirect, suits our system because the relevant structural changes occur inside the sandwiched device architecture. The symmetry of the *I*–*V* curve suggested a molecular junction with nearly symmetric contacts, consistent with the Ag/BDT/Ag configuration from our DFT calculations.^60,61^ To obtain more quantitative information, namely, the effective barrier height, electrode separation, and junction cross-sectional area, we fitted the *I*–*V* curve using the Simmons model^25,59^ assuming symmetric contacts (Figure 4(a)). Considering a simple rectangular potential, the tunneling current is given by eq 1:

1 $$I=\frac{eA}{2\pi hd^{2}}\left\{\left(\phi_{0}-\frac{eV}{2}\right)\;\exp\left[-\frac{4\pi d}{h}\sqrt{2m\left(\phi_{0}-\frac{eV}{2}\right)}\right]-\left(\phi_{0}+\frac{eV}{2}\right)\;\exp\left[-\frac{4\pi d}{h}\sqrt{2m\left(\phi_{0}+\frac{eV}{2}\right)}\right]\right\}$$

where ϕ_0_, *d*, and *A* denote the effective barrier height, electrode separation, and junction cross-sectional area, respectively (Figure 4(a)). From the fitting to the typical *I*–*V* curve (Figure 4(b)), the estimated tunnel barrier (ϕ_0_), distance (*d*), and cross-sectional area (*A*) were 0.27 eV, 1.2 nm, and 5.3 nm^2^, respectively. The fitting results were averaged, and the obtained ϕ_0_, *d*, and *A* were 0.25 ± 0.05 eV, 1.2 ± 0.1 nm, and 6 ± 3 nm^2^, respectively. The ϕ_0_ of 0.25 eV was equivalent to that observed in the BDT molecular junction^62^ but much smaller than that of the OFF state of a Ta_2_O_5_ atomic switch in vacuum, which is approximately 1 eV.^25^ The distance is equivalent to the length of the BDT monolayer.^63^ Considering the SERS and XPS spectra, the fitting results indicated that electron transport occurred through the BDT monolayer. The estimated cross-section provided approximately 22 molecules as the occupation area of BDT in the SAM.^64^ Considering the symmetry of the *I*–*V* curve and the above-mentioned fitting parameters, we reasonably conclude that the Ag filament is electrically connected to the BDT layer. Together with the conductance analysis findings, these results verified the formation of the Ag/BDT/Ag junction in the H state.

**Figure 4:**
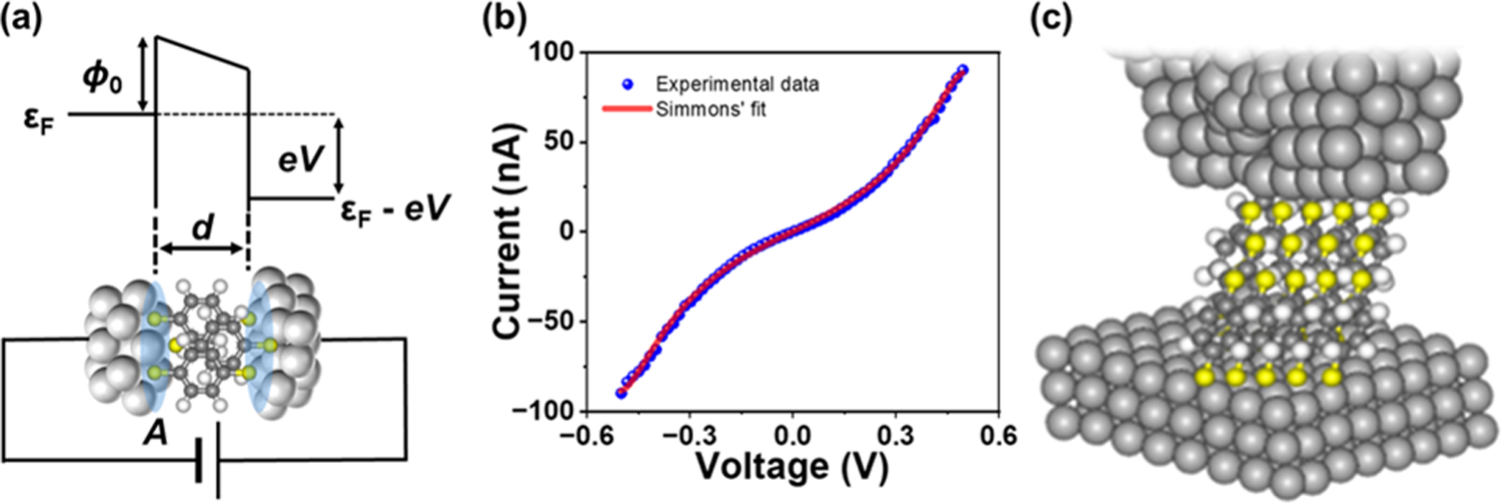
(a) Schematic diagram of electron transport according to the Simmons model. For clarity, electron tunneling via the lowest unoccupied molecular orbital is illustrated here; the same concept applies to hole tunneling mediated by the highest occupied molecular orbital. (b) Typical *I*–*V* characteristics in the H state of the atomic switch with the BDT layer. The blue dots and red solid curve represent the experimental data and a fitted curve with the Simmons model, respectively. (c) Schematic image of the Ag/BDT/Ag junction in the atomic switch with the BDT layer.

In summary, we demonstrated a technique for connecting an atomic switch to a molecular layer on an electrode. The *I*–*V* response of the atomic switch with the BDT layer showed nonvolatile behavior, with H and L states. Supported by DFT calculations, a comparison of the conductance values indicated that the BDT layer was homogeneously connected with Ag. Analysis of the *I*–*V* curve using the Simmons model revealed that the Ag filament was connected to the BDT molecules. Therefore, our method effectively connects a microscale electrode and a nanoscale molecular layer, paving the way for fabricating electronics that leverage the functionalities of molecules.

## Supplementary Material



## Notes

Information about the XPS measurements, SERS measurements, typical *I*–*V* characteristics of the atomic switch without BDT, transition of conductance during *I*–*V* measurements, and theoretical calculations (PDF)
