# Supplementary material for: Electrochemical Wiring of a Metal Nanofilament to Form a Molecular Junction
Source: J Phys Chem Lett. 2026 May 25;17(22):6176–82. doi: 10.1021/acs.jpclett.6c00582 (PMC13244551; doi:10.1021/acs.jpclett.6c00582)
Supplement: Supplementary file 1 [file jz6c00582_si_001.pdf]

Supporting Information

Electrochemical Wiring of a Metal Nanofilament to form a Molecular Junction

Sekito Nishimuro,[1] Tohru Tsuruoka,[2,] Tatsuhiko Ohto,[3,*] Koya Akashi,[1] Tomoaki Nishino,[4]*

Kazuya Terabe,[2] Satoshi Kaneko[1,]*

[1]Department of Materials Science and Engineering, School of Materials and Chemical Technology, Institute of Science Tokyo, 2-12-1, Ookayama, Meguro-ku, Tokyo 152-8550, Japan.
[2] Research Center for Materials Nanoarchitectonics (MANA), National Institute for Materials Science (NIMS), 1-1, Namiki, Tsukuba, Ibaraki 305-0044, Japan.

[3]Graduate School of Engineering, Nagoya University, Furo-cho, Chikusa-ku, Nagoya, 464-8603 Aichi, Japan.

[4]Department of Chemistry, School of Science, Institute of Science Tokyo, 2-12-1, Ookayama, Meguro-ku, Tokyo 152-8550, Japan.

***Corresponding authors:**

E-mail: TSURUOKA.Tohru@nims.go.jp (Tohru Tsuruoka)
E-mail: ohto@nagoya-u.jp (Tatsuhiko Ohto)
E-mail: kaneko.s@mct.isct.ac.jp (Satoshi Kaneko)

Table of Contents

S1. XPS Measurements......2
S2. SERS Measurements......4
S3. Typical *I–V* Characteristics of Atomic Switch without BDT......6
S4. Transition of Conductance during *I–V* Measurements......7
S5. Theoretical Calculations......8
References......9

S1. XPS Measurements

X-ray photoelectron spectroscopy (XPS) measurements were performed using a spectrometer (JPS-9010, JEOL, Japan). The X-ray source was monochromatized AlKα, and the acceleration voltage and emission current were 12 kV and 25 mA, respectively. In addition, the take-off angle was set to 75° in an ultrahigh vacuum of 9.0×10^{-8} Pa. The Pt 4f, S 2p, and C 1s spectra were integrated within the 15–20 eV range with a pass energy of 50 eV; step size of 0.100 eV; integration time of 100 ms; and scan numbers of 5, 300, and 5, respectively.

The spectral background was subtracted from all spectra following the Shirley method.[1] The binding energy was calibrated by the $4f_{7/2}$ state of Pt as follows. The two peaks of the Pt 4f states originating from $4f_{7/2}$ and $4f_{5/2}$ were fitted using two Gaussian functions (Fig. S1).[2] Then, the binding energy of the obtained spectra was calibrated by setting the peak center of the $4f_{7/2}$ state to 71.1 eV.[3]

After binding energy calibration, the C 1s and S 2p states were studied. First, the C 1s spectrum was fitted using two Gaussians (Fig. S2(b)). The peak centers of the two Gaussians were approximately 284.3 and 285.3 eV. The ratio of the areas of the two peaks was 2:1. The binding energy and peak ratio agreed well with the states attributed to the C directly connecting to the S atom and the other aromatic C.[4]

Then, the S2p states were fitted using the two Gaussians considering the two states of the S atom (Fig. S2(c)). The first was the S atom connected to the Pt surface (S–Pt), and the other was a free thiol (S–H), as derived in Eq. (S2).[5,6]

$$I = \left[2\frac{Ae^{-\frac{4\ln(2)(E-E_{c1})^2}{w^2}}}{w\sqrt{\frac{\pi}{4\ln(2)}}} + \frac{Ae^{-\frac{4\ln(2)(E-E_{c1}-1.2)^2}{w^2}}}{w\sqrt{\frac{\pi}{4\ln(2)}}} \right] + \left[2\frac{Ae^{-\frac{4\ln(2)(E-E_{c2})^2}{w^2}}}{w\sqrt{\frac{\pi}{4\ln(2)}}} + \frac{Ae^{-\frac{4\ln(2)(E-E_{c2}-1.2)^2}{w^2}}}{w\sqrt{\frac{\pi}{4\ln(2)}}} \right], \text{(S2)}$$

where I is the photoelectron intensity, E is the binding energy, E_{ci} is the peak center of the binding energy for the P_i state, and w is the full width at half maximum. The area of the peak of S $2p_{3/2}$

was double that of the $2p_{1/2}$ peak, and the energy splitting caused by the spin–orbit interaction was set to 1.2 eV, following previous papers.[5,6] The peak center values of the $2p_{3/2}$ state of P_1 and P_2 were approximately 162.0 ± 0.4 and 163.4 ± 0.2 eV, respectively. The binding energy of the $2p_{3/2}$ state when S is chemisorbed on a metal surface is generally 161.5–162.1 eV.[7,8] The peak center of P_1 was consistent with that of a chemisorbed thiol group.[9,10] The peak center of P_2 was compatible with the binding energy of the unbound S group on the metallic surface (163.5 eV), such as H–S bonds.[6,8] Therefore, the 162.0 and 163.4 eV peak center values were attributed to Pt–S and H–S bonds, respectively, confirming Pt–S bond formation.

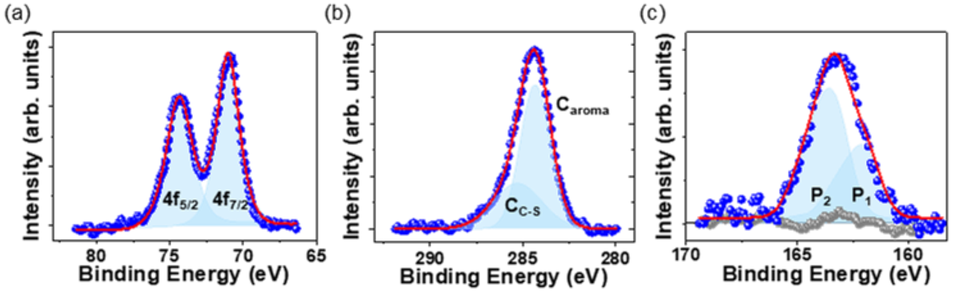

Figure S1. High-resolution XPS spectra of (a) Pt 4f, (b) C 1s, and (c) S 2p core levels from BDT SAM on Pt (raw data, blue dots; peak components, light blue areas; total fit, solid red curve).

S2. SERS Measurements

After the BDT layer was fabricated on the bottom Pt electrodes, a suspension of Au nanoparticles was deposited on BDT and dried. The gap plasmon between the Pt surface and Au nanoparticles enabled the detection of the Raman signal of the BDT monolayer.[11] Raman spectra were acquired using a confocal Raman microscope (NRS-4500, JASCO, Japan). The wavelength and intensity of the excitation laser were 785 nm and 2.4 mW, respectively. Data acquisition time was 500 s. The peak value was derived from the Lorentz function after background subtraction through asymmetric least squares smoothing.

Figure S2 and Table S1 show the SERS spectrum and vibrational assignment of the AuNP/BDT/Pt substrate. The SERS spectrum shows major peaks at 731, 1061, 1178, and 1557 cm^{-1}, consistent with previous findings.[11,12] These peaks corresponded to v_{7a} (C–S stretching mode), v_1 (ring breathing mode), v_{9a} (C–H bending mode), and v_{8a} (C=C stretching vibrational mode), respectively.

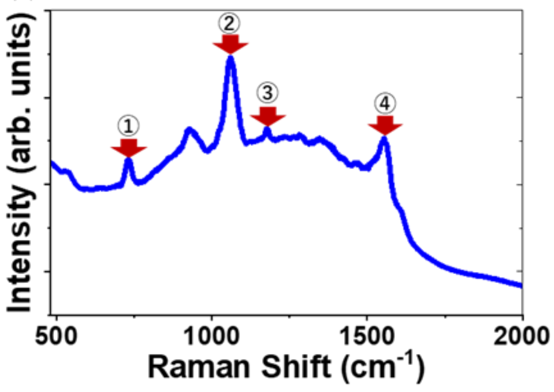

Figure S2. SERS spectrum of AuNP/BDT/Pt.

Table S1. Vibrational Assignment of BDT

No.	SERS (cm^1)	Assignment
1	731	νCS, 7a (a_1)
2	1061	νCH + νCS + νCC, 1 (a_1)
3	1178	δCH, 9a (a_1)
4	1557	νCC, 8a (a_1)

S3 Typical *I–V* Characteristics of Atomic Switch Without BDT

I– V measurements were performed under ambient conditions using a commercial probe station (Micro-Tech, Japan) equipped with a source measure unit (Keithley 2612A, Tektronix, USA). The bottom Pt electrode was electrically grounded. A compliance current (~0.1 mA) was applied to prevent destructive device breakdown and ensure nonvolatile switching under ambient conditions. Figures S3(a) and S3(b) show the typical electrical characteristics of the atomic switch without BDT, which are consistent with previous reports.[13–15] The conductance of the ON state was 9 G_0, corresponding to a metallic quantum point contact.[16,17] Thus, the Ag nanofilament was connected to the Pt electrode (Fig. S3(c)).

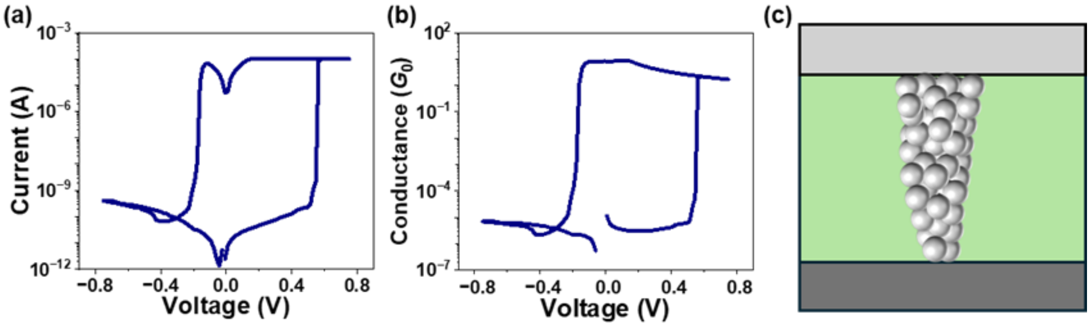

Figure S3. Atomic switch without BDT. (a) Typical *I–V* characteristics. (b) Typical *G–V* characteristics. (c) Schematic image of Ag nanofilament connected to Pt electrode.

S4. Transition of Conductance during *I–V* Measurements

The time course of the conductance behavior was investigated by plotting conductance as a function of the sweep cycle (Fig. S4(a)). G_{forward} is the conductance at 100 mV during the forward sweep, and G_{backward} is the conductance at 100 mV during the backward sweep (Fig. S4(b)). G_{backward} showed a transient increase around cycle 15 and soon decreased to approximately 100 μG_0. It then gradually increased around cycle 80 and finally stabilized at a value identical to the H-state conductance. Similarly, G_{forward} increased in cycle 16 and immediately decreased, in synchrony with G_{backward}. Subsequently, conductance remained nearly constant at a value identical to the L-state value.

The cycle-by-cycle increase in G_{backward} around cycle 80 is likely due to the ion motion in the Ta_2O_5 layer. Several cycles are required to form a continuous filament.[18,19] A thicker filament can be generated by applying additional bias, thus increasing filament conductance.[20,21] Therefore, the change in G_{backward} was attributed to the cycle-by-cycle growth of the conductive filament. Contact geometries can modulate the conductance of a metal/molecule/metal interface, but conductance is randomly modulated in this case.[22-24] The transient conductance increase observed over cycles 15–50 may correspond to the formation of a Ag/BDT/Pt junction with an incomplete filament. As mentioned in the main text, the Ag/BDT/Pt junction is expected to be more conductive than Ag/BDT/Ag. The gradual growth of a Ag cluster on the bottom electrode may reduce conductance; however, further investigation using complementary methods is required because the device's cross-sectional geometry cannot be directly observed.

The behavior of G_{forward} can also be explained by the ion motion in Ta_2O_5. The L-state value around cycle 80 is higher than the initial G_{forward} value, indicating the presence of residual Ag clusters after the bias sweep.

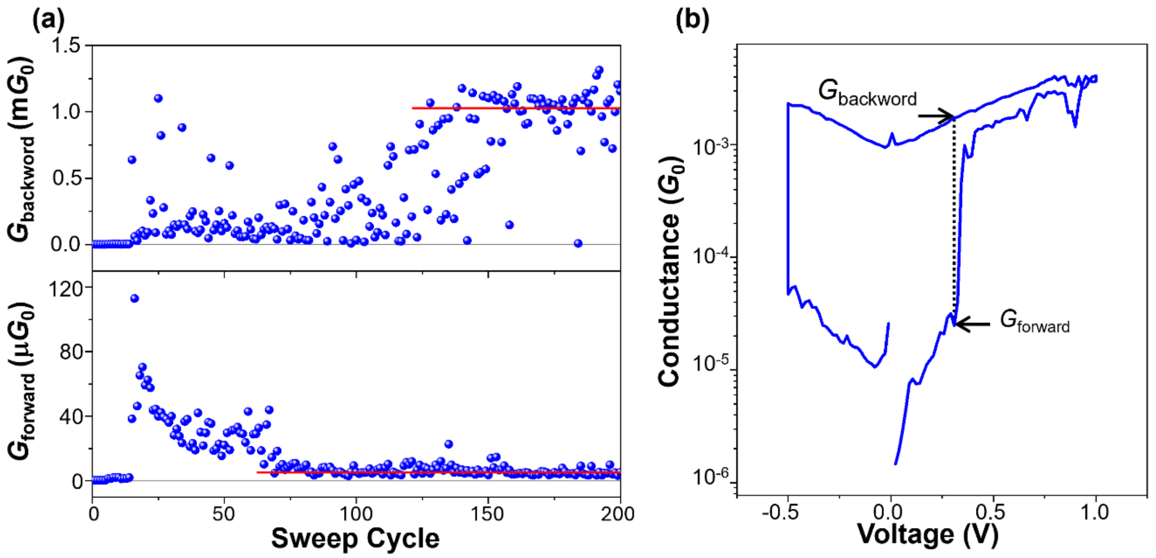

Figure S4. (a) Conductance trace of ON state at 100 mV, showing backward (G_{backward}) and forward (G_{forward}) sweep directions. The red lines indicate the modal conductance values of the H and L states for G_{backward} and G_{forward}, respectively. (b) G–V curve in cycle 178.

S5. Theoretical Calculations

The molecular transport properties were calculated using the SMEAGOL code,[25–27] which is based on the SIESTA program.[28] SIESTA-SMEAGOL adopts the nonequilibrium Green's function (NEGF) combined with DFT (Fig. S5). Single-zeta plus polarization and double-zeta plus polarization basis sets were used for the electrode and other atoms, respectively. Additionally, we used the Perdew–Burke–Ernzerhof exchange-correlation functional. The electrode was modeled as a Ag(111) or Pt(111) slab with p(5×5) periodicity. Then, *k* points were sampled using a uniform 2 × 2 × 1 grid. The lattice mismatch between Pt and Ag was resolved to construct heteroelectrode junction structures; their fcc lattice constants are 3.923 and 4.086 Å, respectively. Because Pt has a partially occupied *d*-band, which is sensitive to the lattice structure, we used Ag electrodes with Pt's lattice parameter in all calculations.

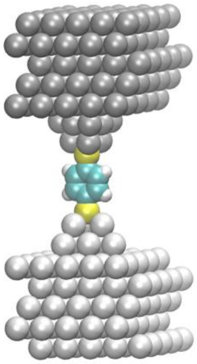

Figure S5. Pt/Ag junction structure used for NEGF-DFT calculations.

References

(1) Shirley, D. A. High-Resolution X-Ray Photoemission Spectrum of the Valence Bands of Gold. *Phys. Rev. B* **1972**, *5*, 4709–4714.

(2) Choi, M.-J.; Park, H.; Engelhard, M. H.; Li, D.; Sushko, P. V.; Du, Y. Reevaluation of XPS Pt 4f Peak Fitting: Ti 3s Plasmon Peak Interference and Pt Metallic Peak Asymmetry in Pt@TiO2 System. *J. Vac. Sci. Technol. A* **2024**, *42*, 063209.

(3) Petrovykh, D. Y.; Kimura-Suda, H.; Opdahl, A.; Richter, L. J.; Tarlov, M. J.; Whitman, L. J. Alkanethiols on Platinum: Multicomponent Self-Assembled Monolayers. *Langmuir* **2006**, *22*, 2578–2587.

(4) Whelan, C. M.; Smyth, M. R.; Barnes, C. J. HREELS, XPS, and Electrochemical Study of Benzenethiol Adsorption on Au(111). *Langmuir* **1999**, *15*, 116–126.

(5) Aliganga, A. K. A.; Lieberwirth, I.; Glasser, G.; Duwez, A.-S.; Sun, Y.; Mittler, S. Fabrication of Equally Oriented Pancake Shaped Gold Nanoparticles by SAM-Templated OMCVD and Their Optical Response. *Org. Electron.* **2007**, *8*, 161–174.

(6) Hamoudi, H.; Prato, M.; Dablemont, C.; Cavalleri, O.; Canepa, M.; Esaulov, V. A. Self-Assembly of 1,4-Benzenedimethanethiol Self-Assembled Monolayers on Gold. *Langmuir* **2010**, *26*, 7242–7247.

(7) Castner, D. G.; Hinds, K.; Grainger, D. W. X-Ray Photoelectron Spectroscopy Sulfur 2p Study of Organic Thiol and Disulfide Binding Interactions with Gold Surfaces. *Langmuir* **1996**, *12*, 5083–5086.

(8) Li, Z.; Chang, S.-C.; Williams, R. S. Self-Assembly of Alkanethiol Molecules onto Platinum and Platinum Oxide Surfaces. *Langmuir* **2003**, *19*, 6744–6749.

(9) Pugmire, D. L.; Tarlov, M. J.; Van Zee, R. D.; Naciri, J. Structure of 1,4-Benzenedimethanethiol Self-Assembled Monolayers on Gold Grown by Solution and Vapor Techniques. *Langmuir* **2003**, *19*, 3720–3726.

(10) Li, Z.; Lieberman, M.; Hill, W. XPS and SERS Study of Silicon Phthalocyanine Monolayers: Umbrella vs Octopus Design Strategies for Formation of Oriented SAMs. *Langmuir* **2001**, *17*, 4887–4894.

(11) Suzuki, S.; Kaneko, S.; Fujii, S.; Marqués-González, S.; Nishino, T.; Kiguchi, M. Effect of the Molecule–Metal Interface on the Surface-Enhanced Raman Scattering of 1,4-Benzenedithiol. *J. Phys. Chem. C* **2016**, *120*, 1038–1042.

(12) Joo, S. W.; Han, S. W.; Kim, K. Adsorption of 1,4-Benzenedithiol on Gold and Silver Surfaces: Surface-Enhanced Raman Scattering Study. *J. Colloid Interface Sci.* **2001**, *240*, 391–399.

(13) Tsuruoka, T.; Terabe, K.; Hasegawa, T.; Aono, M. Forming and Switching Mechanisms of a Cation-Migration-Based Oxide Resistive Memory. *Nanotechnology* **2010**, *21*, 425205.

(14) Tsuruoka, T.; Terabe, K.; Hasegawa, T.; Valov, I.; Waser, R.; Aono, M. Effects of Moisture on the Switching Characteristics of Oxide-Based, Gapless-Type Atomic Switches. *Adv. Funct. Mater.* **2012**, *22*, 70–77.

(15) Aiba, A.; Kaneko, S.; Tsuruoka, T.; Terabe, K.; Kiguchi, M.; Nishino, T. Effects of Water Adsorption on Conductive Filaments of a Ta2O5 Atomic Switch Investigated by Nondestructive Electrical Measurements. *Appl. Phys. Lett.* **2020**, *117*, 233104.

(16) Terabe, K.; Hasegawa, T.; Nakayama, T.; Aono, M. Quantized Conductance Atomic Switch. *Nature* **2005**, *433*, 47–50.

(17) Tsuruoka, T.; Hasegawa, T.; Terabe, K.; Aono, M. Conductance Quantization and Synaptic Behavior in a Ta_2O_5 -Based Atomic Switch. *Nanotechnology* **2012**, *23*, 435705.

(18) Ohno, T.; Hasegawa, T.; Tsuruoka, T. *et al.* Short-term plasticity and long-term potentiation mimicked in single inorganic synapses. *Nat. Mater.* **2011**, 10, 591–595.

(19) Sun, H.; Liu, Q.; Li, C.; Long, S.; Lv, H.; Bi, C.; Huo, Z.; Ling, L.; Liu, M. Direct observation of conversion between threshold switching and memory switching induced by conductive filament morphology. *Adv. Func. Mater.* **2014**, 24, 5679-5686.

(20) Li, Y.; Zhang, M.; Long, S. *et al.* Investigation on the Conductive Filament Growth Dynamics in Resistive Switching Memory via a Universal Monte Carlo Simulator. *Sci. Rep.* **2017**, **7**, 11204.

(21) Srilimkaew, O.; Azhari, S.; Banerjee, D.; Tanaka, H. Short-term and long-term memory functionality of a brain-like device built from nanoparticle atomic switch networks. *Adv. Electro. Mater.* **2024**, 10, 2400360.

(22) Basch, H.; Cohen, R.; Ratner, M. A. Interface geometry and molecular junction conductance: geometric fluctuation and stochastic switching. *Nano Lett.* **2005**, 5, 9, 1668–1675.

(23) Néel, N.; Kröger, J.; Berndt, R. Two-level conductance fluctuations of a single-molecule junction *Nano Lett.* **2011**, 11, 9, 3593–3596.

(24) Kim, H. S.; Kim. Y.-H. Conformational and conductance fluctuations in a single-molecule junction: Multiscale computational study. *Phys. Rev. B* **2010**, 82, 075412.

(25) Rocha, A. R.; García-Suárez, V. M.; Bailey, S.; Lambert, C.; Ferrer, J.; Sanvito, S. Spin and Molecular Electronics in Atomically Generated Orbital Landscapes. *Phys. Rev. B* **2006**, *73*, 085414.

(26) Rungger, I.; Sanvito, S. Algorithm for the Construction of Self-Energies for Electronic Transport Calculations Based on Singularity Elimination and Singular Value Decomposition. *Phys. Rev. B* **2008**, *78*, 035407.

(27) Ohto, T.; Rungger, I.; Yamashita, K.; Nakamura, H.; Sanvito, S. *Ab Initio* Theory for Current-Induced Molecular Switching: Melamine on Cu(001). *Phys. Rev. B* **2013**, *87*, 205439.

(28) José M Soler; Emilio Artacho; Julian D Gale; Alberto García; Javier Junquera; Pablo Ordejón; Daniel Sánchez-Portal. The SIESTA Method for Ab Initio Order-N Materials Simulation. *J. Phys. Condens. Matter* **2002**, *14*, 2745.
